# A Systematic Review of SMART Implantable Devices for Spinal Implants: Current Insights and Future Trends

**DOI:** 10.3390/s26092729

**Published:** 2026-04-28

**Authors:** Mohsen Khodaee, Anna Schuler, Tobias Götschi, Taekwang Jang, Mazda Farshad, Jonas Widmer

**Affiliations:** 1 Department of Orthopedic Surgery, Spine Biomechanics, Balgrist University Hospital, University of Zurich, 8008 Zurich, Switzerland; mkhodaee@ethz.ch (M.K.); anna.schuler@balgrist.ch (A.S.); jonas.widmer@hest.ethz.ch (J.W.); 2 Department of Information Technology and Electrical Engineering, Energy-Efficient Circuits and Intelligent Systems, ETH Zurich, 8092 Zurich, Switzerland; tkjang@ethz.ch; 3 Balgrist University Hospital, University Spine Center Zurich, University of Zurich, 8008 Zurich, Switzerland; mazda.farshad@balgrist.ch; 4 Department of Orthopedics, Balgrist University Hospital, University of Zurich, 8008 Zurich, Switzerland

**Keywords:** SMART implant, spinal fusion, sensor, telemetry, spinal surgery

## Abstract

(1) Background: SMART spinal implants combine biomechanical stabilization with embedded sensors for continuous in vivo monitoring of spinal loading and implant behaviour. This systematic review summarizes current SMART implant technologies in spinal surgery and evaluates their potential clinical applications. (2) Methods: A structured literature search was conducted in PubMed and Scopus in December 2025. Two independent reviewers screened studies using predefined criteria, with data extracted on implant design, sensor type, study model, and application; risk of bias was assessed using the Office of Health Assessment and Translation tool. (3) Results: Thirty-four studies met inclusion criteria, including sensor-integrated posterior rods and fixators (*n* = 16), vertebral body replacements (*n* = 6), intervertebral cages or disc space sensors (*n* = 7), and other configurations (*n* = 5). Devices were tested in human, cadaveric, and animal models. Most systems used strain-based sensors to quantify implant loading, while few employed accelerometers or pressure sensors. Reported results demonstrated activity- and posture-dependent load changes, and several studies indicated potential for monitoring spinal fusion progression by monitoring load trends. (4) Conclusions: Overall, SMART spinal implants primarily support biomechanical monitoring and show promise for real-time assessment of implant performance, though further studies correlating sensor data with clinical outcomes are required.

## 1. Introduction

In recent years, the field of biomedical engineering and orthopedics has seen significant advancements in the development of implantable SMART (Self-Monitoring, Analysis, and Reporting Technology) devices [1,2,3,4,5]. In this review, SMART implants are defined as implantable devices that incorporate integrated sensing elements capable of measuring biomechanical or physiological parameters in vivo, combined with mechanisms for data acquisition and transmission that enable external monitoring. Unlike conventional orthopedic implants, which serve purely structural or supportive functions, SMART implants integrate sensing, data acquisition and processing, and wireless communication technologies to provide real-time monitoring of biomechanical and physiological parameters. These systems have the potential to bring about a paradigm shift in orthopedic and spinal surgery, enabling enhanced postoperative monitoring, early detection of complications, and personalized treatment strategies [6].

Spinal surgery, particularly procedures involving spinal fixation, disc replacement, and vertebral stabilization, could greatly benefit from the integration of SMART implants. Traditional implants, while effective in stabilizing the spine, often require regular and labour-intensive follow-up assessments and imaging to monitor long-term stability and patient recovery. The advent of SMART implants introduces an approach that facilitates continuous in vivo monitoring, optimizing patient care and potentially reducing the need for repeated surgical interventions.

Several research efforts have demonstrated the feasibility of embedding sensing systems into spinal implants to monitor parameters such as strain, load distribution, and motion [4,7,8,9,10,11,12,13]. Initial developments largely centred on benchtop validation and ex vivo testing [14], with limited translation to clinical settings. More recently, advancements in sensor miniaturization, wireless telemetry, and energy management have led to more reliable and implantable prototypes, including systems based on piezoelectric, micro-electro-mechanical systems (MEMS), and capacitive technologies. Related advances in flexible and miniaturized biomedical sensing technologies have also emerged from recent developments in materials science and sensor engineering [15,16,17]. However, issues such as power consumption, data transmission reliability, biocompatibility, and long-term durability continue to pose challenges for routine clinical use.

While the concept of SMART implants has been described in broader orthopedic contexts [6,14,18,19,20], the current review focuses on SMART or telemeterized implants designed for applications in spinal surgery. Following the PRISMA reporting guidelines [21], we aim to systematically assess the available evidence on SMART spinal implants to evaluate their clinical relevance, technological maturity and potential for widespread adoption.

## 2. Methods

### 2.1. Search Strategy

A systematic search was conducted using the PubMed and Scopus databases following the Preferred Reporting Items for Systematic Reviews and Meta-Analyses (PRISMA) guidelines. The search query used was as follows:

(spinal OR spine OR vertebral) AND (implant OR implantable OR prosthesis OR instrumentation) AND (smart OR sensor OR accelerometer OR strain-gauge OR telemetry OR thermometer) AND (“monitor” OR “monitoring” OR “detection” OR “healing” OR “fusion” OR “complication” OR “pseudoarthrosis” OR “infection” OR “subsidence” OR “failure”). The search was conducted across all time periods available in the databases, with no restrictions on publication date.

### 2.2. Selection Criteria

Two authors (M.K. and A.S.) screened the articles independently based on the research question: “What smart implantable devices have been developed for medical use in the human spine, focusing on monitoring healing and detecting complications, and intended for clinical implementation rather than solely for research purposes?”

Studies were considered eligible if they were peer-reviewed primary studies focusing on smart spinal implants, excluding devices for other body parts. Only implants designed for in vivo human use or in vivo animal studies demonstrating clear potential for human application were included. Eligible devices had to incorporate at least one sensor, such as a strain gauge, accelerometer, or thermometer, and be specifically designed for monitoring healing or detecting complications, including pseudoarthrosis, screw loosening, cage subsidence, or implant failure.

Articles were excluded if they were not in English, constituted secondary evidence such as reviews, meta-analyses, book chapters, or commentaries, or did not focus on the spine, such as studies on general orthopedic implants. Studies addressing spinal cord injury, pain, or rehabilitation rather than spinal implants were also excluded. Additional studies were excluded if the implants did not incorporate smart technology, meaning they lacked integrated sensors. Studies focusing primarily on surgical tools, procedural techniques, or complications, such as screw insertion tools or trajectory planning, were also not considered. Additionally, in vitro or cadaveric studies without clear in vivo application potential, studies describing non-implanted devices such as wearables or imaging-based methods, and those unrelated to orthopedic implants were not considered. Studies with mixed experimental designs were included if they contained relevant in vivo human or animal data.

### 2.3. Data Management

The search results were imported into Zotero, where the two reviewers independently categorized the papers as either included or assigned them to one of the predefined exclusion categories. Discrepancies were identified using MATLAB 2022a and subsequently discussed in a consensus meeting.

### 2.4. Risk of Bias Evaluation

The risk of bias for studies included in the primary analysis was assessed using the Office of Health Assessment and Translation (OHAT) risk of bias tool, which is designed for both human and animal research. Eligible studies were evaluated on a four-point scale, ranging from low to high risk of bias, based on 11 criteria across seven domains: selection, confounding, performance, attrition/exclusion, detection, selective reporting, and other sources of bias. The risk-of-bias assessment was performed independently by two reviewers (M.K. and A.S.), and any discrepancies were resolved through discussion and consensus.

## 3. Results

### 3.1. Included Studies

**Figure 1:**
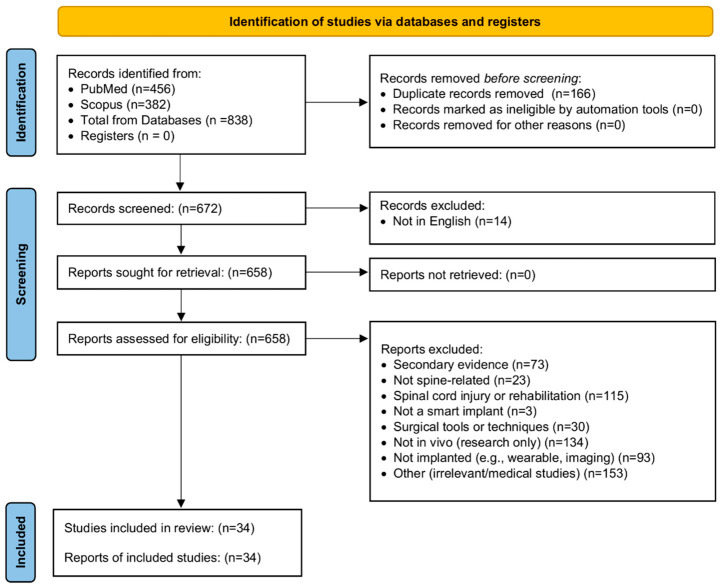
PRISMA flow diagram outlining the systematic process of identification, screening, and inclusion of studies on SMART spinal implants. PRISMA, the Preferred Reporting Items for Systematic Reviews and Meta-Analyses.

Figure 1 illustrates the systematic search process, as well as the inclusion and exclusion criteria applied to the identified papers. A total of 838 papers were retrieved from PubMed and Scopus in December 2025. After an initial screening, duplicate papers and non-English publications were removed, leaving 658 studies for detailed assessment. Following full-text assessment, 34 papers met the eligibility criteria for inclusion in this systematic review. Of these, 21 studies [8,9,10,11,12,22,23,24,25,26,27,28,29,30,31,32,33,34,35,36,37] reported in vivo measurements in human subjects, and 13 studies [38,39,40,41,42,43,44,45,46,47,48,49,50] reported in vivo measurements in animal models. One of the animal studies also included measurements on human cadaveric samples [40].

As shown in Figure 1, several studies were excluded based on specific criteria. A total of 73 studies were identified as systematic reviews or meta-analyses rather than primary evidence. Additionally, 23 studies focused on general orthopedic implants rather than spinal applications, while 115 studies examined spinal cord injury and rehabilitation rather than implant-based monitoring. Three studies were excluded because the investigated implants did not contain measurement sensors. Furthermore, 30 studies were excluded as they primarily addressed surgical tools or techniques rather than implant technologies. Another 134 studies were removed because their measurements were conducted on cadaveric samples without demonstrating potential for in vivo application. Moreover, 93 studies did not involve implanted devices but instead focused on wearable technologies or imaging techniques. Finally, 153 studies were excluded as they were unrelated to SMART implants or orthopedic surgery.

Table 1 and Table 2 summarize the design characteristics and outcome findings of the 34 included studies. Table 1 presents the 21 studies involving in vivo human measurements, while Table 2 presents the 13 studies involving in vivo animal experiments. One of the studies in Table 2 also includes measurements performed on human cadaveric specimens. Among these, 16 investigated sensors or implants mounted on rods or posterior fixators [8,9,10,11,12,24,26,28,32,33,35,37,42,43,45,49], six examined modified vertebral body replacements (VBRs) [25,29,30,31,34,36], and seven focused on intervertebral cages or sensors placed within the intervertebral space [22,38,39,41,44,46,48]. Additionally, one study used a magnetic strip implanted under the patient’s skin [40], while two studies mounted strain gauges directly onto the vertebral lamina [23,27]. Unlike the systematic review by Kim et al. [1], we identified one study that utilized an accelerometer fixed to the lumbar bone [50] and another that employed an intramuscular temperature measurement sensor [47].

Across the included studies, strain gauge-based systems were the most commonly used sensing approach, particularly in posterior fixation devices and vertebral body replacements, primarily for measuring implant load and bending moments [8,9,10,11,12,23,24,26,27,28,29,30,31,32,33,34,35,36,37,42,43,44,48,49]. MEMS-based and pressure-sensing systems were more frequently applied in intervertebral or disc-related applications, where local compressive or pressure-related measurements were of interest [22,38,39,41,46]. In contrast, accelerometer-based and temperature-based approaches were rare and were used to monitor motion-related behaviour [50] or detect infection-related changes [47], respectively.

In addition to differences in implant architecture and sensing mechanisms, studies also varied in their approaches to power supply and data transmission. Many early systems relied on inductive power transfer combined with telemetric data transmission [8,9,10,11,12,24,25,26,28,29,30,31,32,33,34,35,36,37,45,49], enabling continuous measurements without the need for an implanted battery. More recent approaches increasingly employ battery-powered wireless systems, such as radio transmitters [23,27,44,48] or Bluetooth-based communication [42,43]. In contrast, some experimental studies used wired transmission systems or externally powered sensors [22,38,39,46,50], which are mainly suitable for short-term measurements or controlled experimental conditions.

Implants were tested across various vertebral levels, including the cervical (C3–C5) [39,41], thoracic (T5–T12) [23,38], and lumbar spine (L2–L5) [10,12,24,44], with cervical applications being less frequent and thoracic applications rare. The reviewed studies involved human subjects [8,9,10,11,12,22,23,24,25,26,27,28,29,30,31,32,33,34,35,36,37], cadaveric specimens [23,40], and various animal models, including goats [39,41], rats [40], rabbits [47], sheep [42,43,46], dogs [45,49], pigs [38], and baboons [44,48].

The studies show load dynamics across different postures and activities. For instance, higher implant loads were consistently observed during walking, twisting, and lifting [8,23,35], whereas lying in supine position resulted in the lowest forces [33]. Several investigations highlighted the longitudinal monitoring of implant loads before and after spinal fusion procedures [24,29,35], revealing that implant stress can remain elevated or fluctuate postoperatively. Notably, Heumann et al. [43] and Windolf et al. [42] showed how continuous monitoring could identify fusion progression via declining load trends and reduced segment motion over time.

Other innovative approaches included temperature monitoring for infection detection [47], intradiscal pressure measurement [22,46], and biomechanical evaluation of plate stiffness effects on load distribution during fusion [39]. Some implants successfully captured human-like load behaviours in animal models, supporting their translational potential [23,38,44].

### 3.2. Critical Appraisal

The risk of bias in the 34 included studies was assessed using the OHAT assessment tool, as the studies encompassed both animal and human research. The scores for 11 questions across seven bias domains are presented in Table S1. The average score was 28.9 out of 44, indicating moderate methodological limitations, particularly in the domains of selection bias, confounding, and performance bias. In contrast, most studies scored well in other domains, including attrition/exclusion, measurement, and selective reporting bias. It should be noted that certain OHAT domains, particularly those related to randomization, group allocation, and blinding, are primarily tailored to clinical and interventional study designs and may be less directly applicable to engineering or biomechanical investigations. Accordingly, lower scores in these domains should be interpreted within the context of the study design rather than as a direct indication of reduced technical performance of the implant systems.

Each study was assigned a score from 1 to 4 to assess the risk of bias, where 1 indicates “definitely high risk of bias”, 2 indicates “probably high risk of bias”, 3 indicates “probably low risk of bias”, and 4 indicates “definitely low risk of bias”. The score reflects responses to 11 specific questions across seven domains of bias, as follows: Selection bias: (I) Was administered dose or exposure level adequately randomized? (II) Was allocation to study groups adequately concealed? (III) Did selection of study participants result in appropriate comparison groups? Confounding bias: (IV) Did the study design or analysis account for important confounding and modifying variables? Performance bias: (V) Were experimental conditions identical across study groups? (VI) Were the research personnel and human subjects blinded to the study group during the study? Attrition/exclusion bias: (VII) Were outcome data complete without attrition or exclusion from analysis? (VIII) Can we be confident in the exposure characterization? (IX) Can we be confident in the outcome assessment? Selective reporting bias: (X) Were all measured outcomes reported? Other bias: (XI) Were there no other potential threats to internal validity (e.g., statistical methods were appropriate, and researchers adhered to the study protocol)?

## 4. Discussion

### 4.1. Systematic Review

Regular and objective monitoring is a critical component of postoperative care in spinal surgery. As the integration of sensor-equipped devices in surgical domains continues to evolve, SMART implantable technologies are emerging as promising tools for real-time physiological feedback and individualized patient management. In the context of spinal surgery, these devices are able to monitor biomechanical parameters such as axial load, bending moment, and fusion progression, potentially enabling earlier detection of complications like pseudoarthrosis or implant failure, and allowing for data-driven decision-making throughout the recovery process [23,24,43]. However, only a limited number of SMART implants have been developed and tested specifically for spinal applications in vivo [23,38,41,44]. Most studies in this field of research focus primarily on sensor feasibility, data transmission modalities, and biomechanical validation—often in preclinical models—with only a few addressing clinical translation or long-term outcomes [29,39,40]. Furthermore, the synthesis of available studies reveals that human in vivo investigations are predominantly concentrated within a small number of research centres, often involving limited patient cohorts. Although isolated contributions from additional groups exist, the overall clinical evidence base remains structurally narrow.

To further contextualize the strength of this evidence base, the included studies can be broadly categorized into three groups, reflecting both device type and level of evidence. First, established human telemetry studies, predominantly involving instrumented posterior fixation systems and vertebral body replacements, provide direct in vivo measurements and represent the most mature level of clinical evidence. Second, emerging translational studies, often involving intervertebral cages and novel sensor-integrated implant concepts, are primarily evaluated in animal models and demonstrate technological feasibility but remain preclinical in nature. Third, early proof-of-concept devices, such as accelerometer-based motion sensors, temperature sensors for infection monitoring, magnetic sensing concepts, and externally wired pressure or MEMS-based systems, are generally limited to controlled, short-term, or preclinical evaluation. This systematic review consolidates the rapidly evolving literature on SMART spinal implants, emphasizing clinical relevance and real-world applicability from a spine-centred perspective.

### 4.2. Historical Development of SMART Spinal Implants

The evolution of SMART spinal implants has been ongoing for several decades, with one of the first studies on the subject published by Waugh et al. [51], who used instrumented Harrington distraction rods with attached strain gauges to assess forces in scoliosis correction surgery. Further and more extensive investigations followed, such as a study by Elfström et al. [52], involving in vivo telemetric measurements of axial forces in Harrington distraction rods, examining how different body positions influenced implant loading. By the 1990s, semiconductor strain gauges integrated with telemetric units were being successfully applied in human spinal fixators to measure implant loads during various activities [8,9,12]. However, vertebral body replacement devices became more prevalent in the late 2000s and early 2010s [25,29,30,31,34,36]. These studies laid the foundation for next-generation designs incorporating multichannel telemetry [44] and MEMS-based sensors [38,41], enabling more precise measurements with improved biocompatibility. In recent years, advances in technology and the miniaturization of smart implants have renewed interest in posterior fixation measurements, particularly over the last three years [42,43].

The historical development of SMART implants in spine surgery reflects a steady progression from early proof-of-concept studies toward more refined, miniaturized, and clinically viable technologies.

### 4.3. Challenges and Future Directions of SMART Spinal Implants

Despite encouraging advancements, several challenges continue to prevent the integration of SMART spinal implants into standard surgical care. Importantly, many of the systems identified in this review have been evaluated primarily in preclinical models or short-term experimental settings, with relatively limited long-term in vivo data in human patients. Technical limitations such as sensor durability, signal fidelity, miniaturization, and long-term biocompatibility remain aspects of current research [38,40]. In particular, implantable sensing elements must maintain stable performance under repetitive spinal loading over extended implantation periods, which poses challenges for long-term sensor stability and durability. Power supply and data transmission—traditionally reliant on inductive coupling—are evolving toward Bluetooth-enabled systems [42,43], yet the integration into standard clinical practice on a larger scale remains difficult.

In addition, regulatory approval represents an important consideration for future clinical translation, as implantable sensing systems must demonstrate long-term safety, reliability, and clinical benefit before routine clinical adoption can be achieved. Furthermore, variability in patient anatomy and activity levels adds complexity to the interpretation of sensor data, as seen in the fluctuating load measurements across individuals after fusion surgery [27,35]. One potential strategy to address this variability is the use of patient-specific baseline measurements and longitudinal monitoring, where relative changes in implant loading over time may provide more meaningful information than absolute load values [42,43].

Additionally, the biomechanical environment differs between spinal segments [53]. The cervical spine exhibits high mobility and smaller anatomical structures [54], whereas the thoracic spine is relatively stabilized by the rib cage [55], and the lumbar spine is exposed to higher mechanical loads during daily activities [56]. These biomechanical differences may influence the functional requirements of SMART implants and suggest that future systems could benefit from segment-specific designs tailored to the mechanical characteristics of each spinal region.

Future directions include the integration of multimodal sensors capable of detecting not only biomechanical loads but also biological markers for infection or inflammation [47]. In parallel, advances in data processing and analytical methods may support improved interpretation of implant-derived data within broader clinical contexts, while emerging approaches such as machine learning are increasingly being explored in orthopedic research and postoperative monitoring [57].

### 4.4. Study Limitations

While many of the presented systems show promise, limitations remain. The OHAT risk-of-bias assessment indicates that the current evidence base should be interpreted with caution. With an average score of 28.9 out of 44, several studies showed methodological limitations, particularly in the domains of selection bias, confounding, and performance bias. This likely reflects the fact that many investigations in this field are early-stage feasibility or proof-of-concept studies with small cohorts and limited control of confounding factors. Several studies were limited to short-term or preclinical trials [22,38,41], which may not fully reflect long-term physiological responses in humans. Sensor durability, signal stability, and power management—particularly in battery-free telemetry systems [25,40]—remain key engineering challenges. The complexity of in vivo loading conditions, especially the impact of muscle forces [9], also underscores the need for refined biomechanical modelling and data interpretation in future device development.

Importantly, spinal implants are typically used in patients with pre-existing pathologies such as degenerative disease, deformity, or traumatic injury. Consequently, the biomechanical signals recorded by SMART implants reflect a system that has already been altered by disease and surgical intervention, rather than a physiologically normal spine.

Overall, the reviewed SMART spinal implants represent a significant evolution in spinal surgery, offering enhanced diagnostic capabilities and personalized data to support clinical decision-making. However, further research is needed to validate their long-term safety, cost-effectiveness, and clinical utility across broader patient populations.

## 5. Conclusions

SMART spinal implants have been developed to enable monitoring of implant loading, with potential applications in assessing fusion progression, implant failure, and complications such as cage subsidence and screw loosening. Emerging applications, such as temperature-based sensing, may also support the detection of infection. However, clinical translation remains limited, with most studies conducted in preclinical or early in vivo settings with small patient cohorts. This evidence base also shows variability in study design, implant type, and experimental setting, alongside several methodological limitations. In addition, technical barriers, including power supply, data transmission, and biocompatibility, persist. Future research should focus on validating sensor performance in long-term clinical studies and establishing clear correlations between implant-derived data and clinical outcomes.

## Figures and Tables

**Table 1:** Overview of findings from the 21 studies including in vivo human experiments.

Study	Study Population	Study Objectives	Implant Design	Location	Study Outcomes
Rohlmann et al. (1995)	1 male patient	Measurement of loads acting on an internal spinal fixation device in patients with a fractured vertebral body for numerous body positions and activities before and after anterior fusion	Internal spinal fixator Strain gauges Eight-channel telemetric unit with inductive power supply	L3 and L5	Highest loads observed during supine leg lifts. High loads recorded during standing lateral bends, walking, and one-hand carrying. Low loads in lying (recumbent) positions. Post-fusion loads were initially lower than preoperative levels in some cases.
Rohlmann et al. (1995)	1 patient with degenerative instability	Measuring the load in different positions, sitting, standing, walking, turning from supine to lateral	Internal spinal fixator Semiconductor strain gauges Inductively powered	L2–L3 L3–L4	Small load in a relaxed position. Before anterior stabilization, loads were low for sitting, standing, and walking. Sagittal plane bending moment was under 3 Nm. Highest loads in the first 4 weeks occurred when turning from supine to lateral. After anterior stabilization, relaxed lying loads changed slightly. Sitting, standing, and walking had much higher forces and bending moments. Maximum bending moment increased to 5–8 Nm. Sitting loads were not higher than standing.
Graichen et al. (1996)	2 patients (1 with vertebral compression fracture, 1 with degenerative instability)	Body positions: Relaxed lying in a supine, lateral and prone position, sitting and standing were investigated	Internal spinal fixator Semiconductor strain gauges Inductively powered	L4	Before anterior fusion, implant loads were low during lying, sitting, and standing. After surgery, one patient had higher and the other lower loads while sitting and standing. The maximum axial force reached ~370 N, and the maximum bending moment nearly 8 Nm.
Rohlmann et al. (1997)	2 patients (1 with degenerative instability, 1 with vertebral compression fracture)	Loads during walking	Internal spinal fixation device Semiconductor strain gauges Inductively powered	L3–L5 L2–L4	Loads differed strongly before and after anterior fusion as well as between the two patients. Implant loads: Higher in walking than in lying, sitting or standing. Walking speed had little influence on the loads. Staircase walking: Slightly higher loads on the implants than normal walking. Using two crutches reduced loads slightly, a wheeled walker reduced load by 25%.
Rohlmann et al. (1997)	5 lumbar cadaver spines 3 patients (1 with degenerative instability, 1 with L4 compression fracture, 1 with fresh L3 fracture)	Comparing the in vitro and in vivo results for different loading conditions: axial compression (in vitro), standing (in vivo), flexion, extension, lateral bending, and torsion	Modified internal spinal fixator Semiconductor strain gauges Inductive power supply	In vitro: L2/3, L3/4 In vivo: L2/3, L3/4, L4/5	In vitro, flexion and lateral bending sometimes showed tensile axial forces, while patients consistently showed compressive forces. Extension caused extension moments in vitro, but flexion moments in patients. Ignoring muscle forces in vitro can lead to significant differences in fixator load compared to real patient conditions.
Rohlmann et al. (1998)	3 patients (1 with degenerative instability, 2 with compressive vertebral fracture)	Determine the influence of muscle forces in implant loads	Modified internal spinal fixation devices Semiconductor train gauges 8-channel telemetric unit with inductive power supply	L2–4 L3–5 L2–4	Increased implant loads due to muscle contraction in supine, but lower than sitting or standing. Highest implant loads in upright positions. Peak muscle stabilization forces in standing position. Similar implant loads in upside-down and lying positions (muscles offset the tension). Reduced muscle impact on implant loads after anterior fusion.
Rohlmann et al. (1998)	1 patient with degenerative instability	Analyze an unexpected case of pedicle screw breakage three months after implantation	Internal spinal fixator Semiconductor strain gauges Inductively powered	L3–L5	Walking: Flexion bending moments:10 Nm on left and 5 Nm on right fixator. Fatigue fracture: In the upper right pedicle screw. Walking: 360 N on the left and 220 N on the right fixator, varied by about 170 N during each double step. Transverse force components: Less than 25 N. Sitting: Less than 20% lower than during walking. No change: Wearing a Boston overlap brace during the exercises. Lifting both legs ~40° in a supine position increased fixator force by ~370 N, peaking at 500 N in the left implant. Right fixator: 200 and 250 N. Flexion bending moment increased by ~4.7 Nm, peaking at 8.2 Nm in the left fixator. Right implant: 3.2 and 3.4 Nm.
Rohlmann et al. (1999)	10 patients (7 with vertebral compression fracture, 3 with degenerative instability)	Different body positions: Standing, sitting, and lying in a supine, prone, and lateral position	Internal spinal fixation devices Semiconductor strain gauges Inductively powered	L2–L4 L3–L5 T10–T12 T12–L2 T11–L1	Loads are considerably different from patient to patient. Small differences for various lying positions. Flexion bending moments: Significantly higher in upright than in lying body positions. Loads were not higher for sitting than for standing.
Rohlmann et al. (2000)	10 patients (3 with degenerative instability, 7 with vertebral compression fracture)	Measure the loads acting on internal spinal fixators when (i) carrying different loads in one hand, (ii) carrying loads in both hands, and (iii) carrying a load in both hands with the arms raised in anteversion	Modified internal spinal fixator device Strain gauge Telemetric unit and inductive power supply	T11 T12 L1 L3	Carrying weight in one or two hands: Slight load increase vs. standing; lower flexion moment than walking. Axial compression decreased in patients with T11/T12 bridged. Raising arms with weights in both hands: Increased flexion moment in fixators. Fixators bear only a small share of the load.
Rohlmann et al. (2000)	9 patients (6 with vertebral body compression fracture, 3 with degenerative instability)	Comparison of fixator loads before and after anterior interbody fusion in several body positions and activities including standing, walking and lying supine	Modified internal spinal fixator Semiconductor strain gauges Inductive power supply	T11–L1 L3/L4	Implant loads increase after fusion with a bone graft. Distraction in the bridged region raises implant loads in fracture cases and alters loads in degenerative instability. Keeping the lower bridged disc intact results in minimal changes in fixator load post-fusion. Bone grafts alone do not ensure reduced implant loads.
Rohlmann et al. (2000)	10 patients (7 with vertebral compression fracture, 3 with degenerative instability)	20 measuring sessions for different activities, including walking, standing, sitting, lying in the supine position, and lifting an extended leg while in the supine position	Internal spinal fixator Semiconductor strain gauges Inductively powered	L2–L4 L3–L5 T10–T12 T11–L1 T12–L2	High loads even after fusion for most patients. Flexion bending moment even with the body in a relaxed lying position. Average axial force: Between 25 N tensile force and 240 N compressive force. Patients with an L3 or L4: Higher compression forces than patients with T11, T12 or L1. Implant loads decreased after 180 days for only 1 patient and were very low after 1 year. Most absolute values were slightly lower for sitting and slightly higher for walking.
Rohlmann et al. (2001)	10 patients (7 with vertebral compression fracture, 3 with degenerative instability)	Sitting on stool, a stool with a padded wedge, a chair, a physiotherapy ball, a knee stool, and a bench Sitting relaxed and erect on a stool Chair with different inclinations of the backrest Standing up and sitting down	Internal spinal fixation device Semiconductor strain gauges Inductively powered	T11 T12 L1 L3 L4	Minor differences for sitting on different types of seats. Sitting erect: 11% higher implant loads than sitting relaxed. Loads decreased with increasing inclination. Standing up and sitting down: 27% higher compared to sitting.
Szivek et al. (2002)	3 cadaver spines 2 patients	Strain distribution on the thoracic vertebrae during anteroposterior bending and torsion for use with an implantable strain gauge system and miniature radio transmitter	ISOLA construct instrumentation Calcium phosphate ceramic-coated strain gauges attached to the lamina Radio transmitter	T5 T7 T9 T12	The largest and most consistent strain changes after simulated fusion occurred during torsional loading on the laminae beneath a hook. The strain gauges showed excellent bone bonding to the lamina during fusion. Radio telemetry accurately tracked strain magnitudes and strain rates expected in patients.
Szivek et al. (2005)	1 female patient with idiopathic scoliosis	To develop a continuous in vivo monitoring technique for detecting fusion and assessing bone loading during rehabilitation after thoracoscopic anterior release and posterior fusion.	Strain gauges attached to the lamina Subminiature remotely powered radio transmitter	Th12–L2 Th9–11	Left rod strains showed acceptable loading immediately after surgery. Lamina strain changes were largest and most consistent during twisting.
Rohlmann et al. (2008)	3 patients with vertebral compression fracture	Loads on a vertebral body replacement Within the first month postoperatively	Vertebral body replacement Strain gauge Inductively powered	T12–L2 T11–L3	Standing: 150 N. Sitting: 450 N. Flexion: More than 420 N. Elevating both arms in the sagittal plane against a physiotherapist’s resistance: More than 700 N. The highest resultant bending and torsional moments: Less than 4 Nm.
Shahadi et al. (2008)	1 patient with lumbar disc herniation	Different body positions: Lying on back, lying on side, sitting, standing, bending forward, bending backwards, walking, climbing stairs	Pressure sensor inside disc space Wired connection (limited time measurement)	L4–L5	Intradiscal pressure: 10 times lower than the values known from unoperated individuals (lying: up to 130 mmHg, sitting: up to 50 mmHg, standing: up to 450 mmHg. 100 mmHg = 0.013332 MPa).
Rohlmann et al. (2010)	5 patients with vertebral compression fracture	Measure the loads acting on a VBR for single and multi-axis vibration of different intensity levels in different postures (sitting freely, vertical backrest, backrest declined by 25°)	Modified vertebral body replacement Strain gauges Telemetry with inductive power supply	L1 L3	High-intensity, multi-axis vibration raised forces by 89% vs. relaxed sitting. Backrest use and lower intensity reduced implant loads. Car or public transport (with backrest) causes lower loads than walking safely soon after surgery.
Rohlmann et al. (2011)	5 patients with vertebral compression fracture	Sitting on a stool and inclining the upper body from 15° flexion to 10° extension in 5° steps On a chair with adjustable backrest: angles from 108° to 180° On an office chair with seat height between 40 and 60 cm in 5 cm steps Seven seat types tested Various arm positions	Vertebral body replacement Strain gauge Inductively powered	T12–L2 T11–L3 L2–L4	48% increased force for 15° flexion. 19% decreased force for 10° extension. Increasing backrest declination angle: Decreased load. Seat height: Minor effect on load. Compared to sitting on a stool, less loads when sitting on a bench (7%), stool with a padded wedge (9%), a knee stool (19%), a chair (35%), and an office chair (41%). Sitting on a physiotherapy ball increased the loads by 7%.
Srbinoska et al. (2013)	4 patients with compression fracture of a lumbar vertebral body	Correlation between the back shape of the lumbar region and the spinal loads during activities performed in the sagittal plane (lying, standing or sitting)	Vertebral body replacement Strain gauge Inductively powered	T12–L2 T11–L3 L2–L4	Upper body flexion: Average force increase of 285 N and 15° decrease in lordosis angle. Force change when lifting 30 N with one hand: ~190 N on average; 2° change in lordosis angle. Correlation coefficients > 0.6 for exercises with large back shape and load changes, like upper body flexion. Spinal load strongly increases with lordosis angle changes. Strong correlation between lordosis angle and implant force only when angle changes are significant in an upright position.
Rohlmann et al. (2013)	5 patients with vertebral compression fracture	28 measuring sessions for different activities, including standing and walking	Vertebral body replacement Strain gauge Inductively powered	T12–L2 T11–L3 L2–L4	In one patient, forces decreased in the first year but increased over the next four years. In another patient, forces rose in the first months and then declined. In a third patient, forces initially increased, then decreased, while another showed only slight variations. Two patients experienced a strong force drop in the first two postoperative months. Walking forces were on average 100 N or 71% higher than standing forces.
Rohlmann et al. (2014)	5 patients with vertebral compression fracture	Loads on a vertebral body replacement Level and staircase walking The effects of walking speed and using walking aids	Vertebral body replacement Strain gauge Inductively powered	T12–L2 T11–L3 L2–L4	Walking: 171% of standing. Ascending stairs: 265% of standing. Descending stairs: 225% of standing. Walking speed: Strong effect on the implant force. Using a walker: 62% of standing.

**Table 2:** Overview of findings from the 13 studies including in vivo animal experiments.

Study	Study Population	Study Objectives	Implant Design	Implant Location	Study Outcomes
McDonald & Rowell (1976)	7 dogs	Measuring the tensions associated with intraoperative insertion Measuring strain in Dwyer’s implants at the high-tension areas including cable ends or curve apex Measuring the changes to predict interbody fusion	Titanium load cell (between the end screw head and the terminal load of the cable) in Dwyer instrumentation Semiconductor strain gauges Inductively powered telemetry unit	Thoracolumbar or lumbar spine	Cable tension increased postoperative, confirming successful spinal fusion. In vivo and in situ strain measurements matched closely. No tissue reaction or device failure was observed after 8 months.
Shapiro et al. (1978)	7 dogs	Measuring tension in a titanium cable during the Dwyer procedure for scoliosis	Dwyer cable tension monitoring Strain gauge transducer Inductive telemetry system with antenna	Thoracic and lumbar spine	Tension drops immediately after crimping screws. Stress increases tension in early postoperative weeks. After 8–12 months, fusion shows no tension change.
Ledet et al. (2000)	2 baboons	In vivo forces in the lumbar spine using body weight calculations	Interbody implant load cell Strain gauge 16-channel telemetry transmitter	L4–L5	Highest: Strain, 2.8 times body weight. Compressive load: Up to 460 N.
Ferrara et al. (2003)	1 adult male goat	In vivo animal study for biocompatibility assessment of MEMS materials for spinal fusion monitoring	1 MEMS pressure	1 MEMS pressure sensor in C4/5 bone graft, 2 silicon chips in L3/4 disc, 2 pressure sensors in L4/5 bone graft	Good biocompatibility of MEMS materials with no adverse foreign body response.
Ledet et al. (2005)	2 male baboons	Real-time telemetered data were collected from sensor-imbedded implants that were placed in the interbody space of the lumbar spines of two baboons	Intervertebral cage Strain gauges Implantable 16-channel, battery-operated telemetry system	L4/5 intervertebral disc spaces	In vivo implant force measurements had less than 10% error. Lumbar spine loads vary with activity and posture. Max loads exceeded 4 times body weight while flexed sitting in baboons. Force patterns in baboons and humans showed similar trends.
Colloca et al. (2009)	25 sheep	Comparing invasive lumbar bone acceleration to noninvasive displacement responses Assessing single-level disc degeneration effects	Attached to intraosseous pins rigidly fixed to the L1 and L2 Triaxial accelerometers 16-bit data acquisition system (wired)	L1/2 L3	L3 dorsoventral (DV) displacement and L2 acceleration showed a strong linear correlation in both groups. L3 DV displacement was lower in the degenerated group. Mean L2–L1 acceleration transfer dropped from 12.40 to 5.50 m/s^2^ in degeneration.
Glos et al. (2010)	6 pigs	Pressure sensors implanted in the annulus fibrosus of the intervertebral disc for a duration of 8 weeks	MEMS-based piezoresistive pressure sensor dies Copper instrument wires for data transmission	T7/8 and T5/6 intervertebral disc annulus	Two sensors survived the full implantation period. Sensitivity changed by less than 5% pre- and post-implantation. Future improvements could involve smaller, armoured MEMS dies with optimized packaging for longer-term implantation.
Roriz et al. (2014)	1 sheep	To measure the intradiscal pressure signal of an anesthetized sheep under spontaneous breathing To calculate the average resting intradiscal pressure and compare it with available literature data	Intervertebral cage Ultra-miniature fibre optic high-pressure sensor Purpose-built interrogation unit connected to a portable computer	L4/5 IVD	Periodicity of the intradiscal pressure signal was similar to the mean respiratory rate of the animal. Pressure fluctuations ranged between 2.31 and 3.45 bars with a maximum amplitude of 1.14 bars. Thoracic discs in humans, sharing kyphotic curvature with sheep lumbar spines, showed similar resting pressures (2.0–3.4 bar).
Peterson et al. (2018)	10 goats	Interbody loads in goats were measured after ACDF with varying plate stiffness during fusion	Force-sensing spine interbody implant Force load cell (Forsentek) Wired data transmission	C3–C4	Interbody forces in flexion/extension were dynamic. High-stiffness plates led to max forces in extension, low-stiffness in flexion. Fusion reduced interbody load magnitude.
Glassman et al. (2021)	12 rabbits	Local temperature elevation for infection detection 4 groups, with 4 different doses of Staphylococcus aureus, and saline control	Spinal screw-rod Temperature probes (IPTT-300) Passive, battery free (inductive)	L6 transverse process	Infected animals: Statistically significant difference in the scapular control temperature and implant site temperature. Non-infected animals: No temperature difference.
Windolf et al. (2022)	1 Swiss white alpine sheep	Investigate whether continuous implant load measurement can be applied to the spine to monitor spinal fusion progress	Sensors clamped to the fixation rods Strain gauges Bluetooth data transmission to a smartphone	L2/3, L3/4, L2/4	Rod load at euthanasia dropped to 67% (L23) and 30% (L34) of maximum. Operated segments had reduced motion vs. intact segment (L23 and L34 < 1°, L45 = 4.17°). Imaging showed facet joint fusion and bridging bone at both segments.
Shi et al. (2024)	18 rats 9 human cadavers	Real-time monitoring of spinal and joint movements	Flexible magnetic strip External receiver (battery-free)	Subcutaneous paravertebral pocket	High reliability of the implant in monitoring the movement of human body segments. The implant is biocompatible, exhibits excellent functionalities, and can be conveniently implemented in both rats and humans.
Heumann et al. (2024)	3 female sheep	Continuous implant load monitoring as a method for assessing spinal fusion progression over 16 weeks	Sensors clamped to the fixation rods Strain gauges Bluetooth data transmission to a smartphone	L2/3 and L4/5	Implant loads initially increased for four weeks after surgery, then decreased. Nine of twelve sensors plateaued between weeks 7 and 12, indicating fusion completion. CT scans confirmed fusion in all motion segments, showing bilateral trabecular bone formation bridging the excised facet joints. Biomechanical testing showed minimal remnant motion (<0.7°) in all fused segments, confirming successful fusion.

## Data Availability

No new data were created or analyzed in this study. Data sharing is not applicable to this article.

## Supplementary Materials

The following supporting information can be downloaded at: https://www.mdpi.com/article/10.3390/s26092729/s1, Table S1. OHAT risk of bias assessment for 34 included studies.
