# Supplementary material for: A Systematic Review of SMART Implantable Devices for Spinal Implants: Current Insights and Future Trends
Source: Sensors (Basel). 2026 Apr 28;26(9):2729. doi: 10.3390/s26092729 (PMC13165599; doi:10.3390/s26092729)
Supplement: Supplementary file 1 [file sensors-26-02729-s001.zip › sensors-4153137-supplementary.pdf]

Table S1. OHAT risk of bias assessment for 34 included studies.

Study	1	2	3	4	5	6	7	8	9	10	11	Total (out of 44)
McDonald & Rowell (1976)	1	1	2	2	4	1	2	2	2	2	2	21
Shapiro et al. (1978)	1	1	1	2	4	1	3	3	3	3	4	26
Rohlmann et al. (1995)	1	1	1	2	4	1	3	4	4	4	3	28
Rohlmann et al. (1995)	1	1	1	2	4	1	3	3	3	4	4	27
Graichen et al. (1996)	1	1	1	2	4	1	2	3	3	4	4	26
Rohlmann et al. (1997)	1	1	2	2	3	1	3	4	3	4	3	27
Rohlmann et al. (1997)	1	1	1	2	4	1	3	3	3	4	4	27
Rohlmann et al. (1998)	1	1	1	2	4	1	3	3	3	4	4	27
Rohlmann et al. (1998)	1	1	3	3	4	3	3	4	3	4	3	30
Rohlmann et al. (1999)	1	1	2	2	4	1	3	3	4	4	4	29
Rohlmann et al. (2000)	1	1	3	3	4	1	3	4	4	4	3	31
Rohlmann et al. (2000)	1	1	2	2	4	1	3	4	3	4	4	29
Rohlmann et al. (2000)	1	1	1	2	4	1	3	3	3	4	4	27
Rohlmann et al. (2001)	1	1	2	2	4	1	3	4	3	4	4	29
Ledet et al. (2000)	1	1	1	2	4	1	2	3	3	3	4	25
Szivek et al. (2002)	1	1	2	2	4	1	3	4	3	4	3	28
Ferrara et al. (2003)	1	1	2	2	4	1	3	4	3	4	2	27
Ledet et al. (2005)	1	1	2	2	4	1	3	4	3	4	2	27
Szivek et al. (2005)	1	3	3	3	4	3	4	4	4	4	4	37
Rohlmann et al. (2008)	1	1	1	2	4	1	3	3	3	4	4	27
Shahadi et al. (2008)	1	1	1	2	4	1	2	3	3	4	3	25
Colloca et al. (2009)	2	2	2	2	4	2	3	3	3	4	4	31
Glos et al. (2010)	1	2	3	3	4	1	3	4	4	4	3	33
Rohlmann et al. (2010)	3	3	3	3	4	3	4	4	4	4	4	39
Rohlmann et al. (2011)	1	1	1	2	4	1	3	3	3	4	4	27

Srbinoska et al. (2013)	1	2	3	2	4	1	3	4	4	4	3	31
Rohlmann et al. (2013)	1	1	2	2	4	1	3	4	3	4	3	28
Rohlmann et al. (2014)	1	1	1	2	4	1	3	3	3	4	4	27
Roriz et al. (2014)	1	2	3	3	4	3	4	4	4	4	4	36
Peterson et al. (2018)	1	1	2	2	4	1	3	4	4	4	4	30
Glassman et al. (2021)	2	1	2	2	4	1	3	4	4	3	4	30
Windolf et al. (2022)	1	1	1	2	4	1	3	4	4	4	3	28
Shi et al. (2024)	1	1	2	2	4	1	3	4	3	4	3	28
Heumann et al. (2024)	1	2	3	3	4	3	4	4	4	4	4	36

Each study was assigned a score from 1 to 4 to assess the risk of bias, where 1 indicates „definitely high risk of bias“, 2 indicates „probably high risk of bias“, 3 indicates „probably low risk of bias“, and 4 indicates „definitely low risk of bias“. The score reflects responses to 11 specific questions across seven domains of bias, as follows: Selection bias: (I) Was administered dose or exposure level adequately randomized? (II) Was allocation to study groups adequately concealed? (III) Did selection of study participants result in appropriate comparison groups? Confounding bias: (IV) Did the study design or analysis account for important confounding and modifying variables? Performance bias: (V) Were experimental conditions identical across study groups? (VI) Were the research personnel and human subjects blinded to the study group during the study? Attrition/exclusion bias: (VII) Were outcome data complete without attrition or exclusion from analysis? (VIII) Can we be confident in the exposure characterization? (IX) Can we be confident in the outcome assessment? Selective reporting bias: (X) Were all measured outcomes reported? Other bias: (XI) Were there no other potential threats to internal validity (e.g., statistical methods were appropriate, and researchers adhered to the study protocol)?
